# Interactions and Trends of Interleukins, PAI-1, CRP, and TNF-α in Inflammatory Responses during the Perioperative Period of Joint Arthroplasty: Implications for Pain Management—A Narrative Review

**DOI:** 10.3390/jpm14050537

**Published:** 2024-05-17

**Authors:** Arabela-Codruta Cocea, Cristian Ioan Stoica

**Affiliations:** 1 Faculty of Medicine, Doctoral School, “Carol Davila” University of Medicine and Pharmacy, 050474 Bucharest, Romania; 2 Orthopedics, Anaesthesia Intensive Care Unit, Faculty of Medicine, “Carol Davila” University of Medicine and Pharmacy, 050474 Bucharest, Romania; cristian.stoica@umfcd.ro

**Keywords:** interleukin (IL), PAI-1 (plasminogen activator inhibitor-1), TNF-α (tumor necrosis factor-alpha), pain management, surgical outcomes, cytokine interactions, postoperative inflammation, inflammation biomarkers, therapeutic targets

## Abstract

Inflammation during the perioperative period of joint arthroplasty is a critical aspect of patient outcomes, influencing both the pathophysiology of pain and the healing process. This narrative review comprehensively evaluates the roles of specific cytokines and inflammatory biomarkers in this context and their implications for pain management. Inflammatory responses are initiated and propagated by cytokines, which are pivotal in the development of both acute and chronic postoperative pain. Pro-inflammatory cytokines play essential roles in up-regulating the inflammatory response, which, if not adequately controlled, leads to sustained pain and impaired tissue healing. Anti-inflammatory cytokines work to dampen inflammatory responses and promote resolution. Our discussion extends to the genetic and molecular influences on cytokine production, which influence pain perception and recovery rates post-surgery. Furthermore, the role of PAI-1 in modulating inflammation through its impact on the fibrinolytic system highlights its potential as a therapeutic target. The perioperative modulation of these cytokines through various analgesic and anesthetic techniques, including the fascia iliac compartment block, demonstrates a significant reduction in pain and inflammatory markers, thus underscoring the importance of targeted therapeutic strategies. Our analysis suggests that a nuanced understanding of the interplay between pro-inflammatory and anti-inflammatory cytokines is required. Future research should focus on individualized pain management strategies.

## 1. Introduction

Inflammation of the peripheral and central nervous systems is a significant factor in the development and persistence of various pathological states of pain. Specific inflammatory cytokines present in the spinal cord, dorsal root ganglion, injured nerve, and skin have been associated with pain [1,2,3,4,5,6,7,8,9,10,11].

Cytokines are small proteins secreted by cells and have a unique effect on cell interactions and communication. These signaling molecules are responsible for regulating immune responses and inflammation as well as promoting cell growth and differentiation. Inflammatory cytokines are released in response to tissue injury or infection and have the potential to activate nerve fibers, contributing to the development of chronic pain [1].

Understanding the role of cytokines in pain signaling and inflammation is critical for developing new treatments for pain management. By targeting specific cytokines, researchers can potentially develop more effective therapies to reduce pain and improve the quality of life of patients [1].

Interleukins (ILs) play major roles in modulating immune responses in different scenarios, from infectious diseases to pain and the postoperative period. ILs function as both pro-inflammatory and anti-inflammatory ILs and play crucial roles in immune cell activation, tissue repair, and overall balance of the immune system. Pro-inflammatory ILs, such as IL-1β and IL-6, initiate and enhance the immune system, but can lead to tissue damage if overexpressed [12,13,14,15]. Conversely, anti-inflammatory ILs such as IL-10 and TGF-β help limit inflammation and prevent tissue damage, although their excessive activity can cause immune suppression. Overall, the balance between pro-inflammatory and anti-inflammatory ILs is vital for managing immune responses, and this study offers valuable insights [16].

The production of proinflammatory cytokines is primarily attributed to activated macrophages, which induce inflammatory responses. Compelling evidence suggests that specific pro-inflammatory cytokines such as IL-1β, IL-6, and TNF-α play a crucial role in the development and progression of pathological pain [1,12,13,14,15].

This article delves into the crucial roles that interleukins, PAI-1, CRP, TNF-α, and other inflammatory biomarkers play in different scenarios, mainly in the perioperative period of total joint replacement, with a particular emphasis on their pro-inflammatory and anti-inflammatory effects. We will examine different biomarkers that participate in the immune response and their modes of operation. The discussion presented in this chapter also describes several key pro-inflammatory and anti-inflammatory cytokines or other biomarkers, their relationship with pathological pain in animals and human patients, and in the context of total joint replacement. Our aim was to enhance the comprehension of the intricate interplay between the immune system, inflammation, pain, and perioperative period during total joint replacement.

## 2. Pro-Inflammatory Cytokines and Pain

Specific pro-inflammatory cytokines, such as interleukin-1 (IL-1), IL-6, and tumor necrosis factor-α (TNF-α), which are produced mostly by activated macrophages and play a major role in the up-regulation of the inflammatory response, are also involved in the process of pathological pain [1].

### 2.1. Interleukin-6

The IL-6 protein is a multifunctional cytokine that affects a wide range of biological processes. The –174G > C functional genetic polymorphism in IL6 can influence the production and concentration of IL-6. In vitro studies have shown that the polymorphism affects IL-6 mRNA levels, with –174C being associated with lower IL-6 levels and a reduced response to lipopolysaccharide stimulation [17]. These in vitro findings were supported by in vivo observations: healthy 174C allele carriers were found to have significantly lower levels of plasma IL-6 [17].

### 2.2. Interleukin-1β

IL-1β is released during infection, invasion, cell injury, and inflammation from monocytes and macrophages, as well as from non-immune cells, such as endothelial cells and fibroblasts [1,18]. It has also been reported that IL-1β is also expressed in the nociceptive dorsal root ganglion neurons [18]. Following crush injury to the peripheral nerve and trauma, IL-1β expression is upregulated in microglia and astrocytes in the central nervous system (CNS) [19]. Administration of IL-1β through intraperitoneal, intracerebroventricular, or intraplantar routes has been shown to induce hyperalgesia [20,21]. Furthermore, it has been observed that IL-1β enhances the synthesis of substance P and prostaglandin E2 across various neuronal and glial cells [22,23]. IL-1ra, an antagonist specific to the IL-1β receptor, competes with IL-1β for receptor binding, but does not initiate a cellular response, effectively inhibiting IL-1β-induced cellular alterations. Utilizing IL-1ra and additional anti-inflammatory cytokines may mitigate or alleviate inflammatory hyperalgesia [24] and mechanical allodynia [1], resulting from cytokine-driven inflammation and nerve damage.

### 2.3. Tumor Necrosis Factor-α

TNF-α, also known as cachectin, is a key inflammatory cytokine that has been implicated in various pain models. It influences numerous signaling pathways by interacting with two cell surface receptors, TNFR1 and TNFR2. These interactions regulate apoptotic pathways, activate NF-κB for inflammation, and trigger stress-activated protein kinases (SAPKs) [25]. TNF-α receptors are found in both neurons and glial cells. This cytokine is critically involved in both inflammatory and neuropathic hyperalgesia. In adult rat study models, the intraplantar administration of complete Freund’s adjuvant (CFA) leads to a marked increase in TNF-α, IL-1β, and nerve growth factor (NGF) levels in the inflamed paw [26]. Administering anti-TNF-α antiserum prior to CFA exposure significantly postpones the onset of inflammatory hyperalgesia and diminishes IL-1β levels but not NGF levels [26]. Additionally, intraplantar injections of TNF-α result in both mechanical and thermal hyperalgesia [21,27]. TNF-α injection into nerves has been shown to induce Wallerian degeneration and mimic transient behaviors and endoneurial pathologies associated with experimentally induced painful nerve injuries [28]. TNF-binding protein (TNF-BP), which serves as an inhibitor of TNF, is a soluble form of the transmembrane TNF receptor. Systemic administration of TNF-BP effectively reduced hyperalgesia, which is typically observed following lipopolysaccharide (LPS) administration. Moreover, intrathecal delivery of a combination of TNF-BP and an IL-1 antagonist has been shown to alleviate mechanical allodynia in animal models undergoing L5 spinal nerve transection [1].

## 3. Pathological Pain and Glial Activation

Lumbar astrocytes exhibit morphological changes and elevated GFAP expression, which are indicative of an enhanced state of activation, after chronic constriction injury (CCI) in the rat sciatic nerve. This foundational study provides anatomical insights that underscore the involvement of spinal glia in pain modulation. Subsequent research has shown that spinal glia are integrally associated with neural synapses, share neurotransmitter receptors with neurons, and adopt “activated” phenotypes following abnormal or intense neuronal signaling [29,30,31,32,33,34,35]. Astrocytes and microglia possess receptors relevant to pain transmission, such as AMPA, metabotropic glutamate receptors (mGluR), purinergic receptors (P2X4R or P2X7R), and neurokinin 1 receptor (NK1R). These receptors are stimulated by neurotransmitters such as glutamate, ATP, and substance P, which are released from presynaptic terminals [34]. After peripheral nerve damage, neurons secrete chemokines, such as CCL2 [36] and CX3CL1 [36], and other immune factors, including colony-stimulating factor 1 (CSF-1) and ATP [37,38], which robustly activate astrocytes and microglia [39]. These activated glial cells subsequently release pro-inflammatory cytokines (e.g., IL-1β, TNF-α, and IL-6) [40], chemokines (e.g., CCL2) [40,41,42], ATP, excitatory amino acids (EAAs), and nitric oxide (NO) [41,43,44,45,46].

Similarly, dorsal horn neurons in the spinal cord express receptors for these immune mediators, including pro-inflammatory cytokines such as IL-1β [47,48,49,50,51], TNF-α [52,53,54,55,56,57,58], IL-6 [59,60], and IL-17. The activation of these cytokine receptors on neurons influences neuronal function. For instance, pro-inflammatory cytokines such as TNF-α and IL-1β modify synaptic transmission by enhancing excitatory pathways and reducing inhibitory pathways within the spinal cord lamina II [30,61]. Specifically, TNF-α increases the frequency of spontaneous excitatory post-synaptic currents (sEPSC), while IL-6 decreases the frequency of spontaneous inhibitory post-synaptic currents (sIPSC). IL-1β notably increases both the frequency and amplitude of sEPSCs and decreases those of sIPSCs. Furthermore, TNF-α and IL-1β intensify excitatory AMPA- and NMDA-induced currents, whereas IL-1β and IL-6 diminish inhibitory GABA- and glycine-induced currents [61]. Activation of these cytokine receptors amplifies the sensitivity of local neurons and perpetuates a cycle of nociceptive signaling.

## 4. Anti-Inflammatory Cytokines

Anti-inflammatory cytokines are key immunoregulatory molecules that help control the response of pro-inflammatory cytokines and play a crucial role in regulating processes involved in the human immune response. They function together with specific cytokine inhibitors and soluble cytokine receptors to modulate inflammation. Their roles are pivotal both physiologically, in managing inflammation, and pathologically, in systemic inflammatory states. The major anti-inflammatory cytokines include interleukin (IL)-1 receptor antagonists, IL-4, IL-10, IL-11, and IL-13. Additionally, leukemia inhibitory factor, interferon-α, IL-6, and transforming growth factor (TGF)-β can act as either anti-inflammatory or pro-inflammatory cytokines depending on the context. Specific cytokine receptors for IL-1, TNF-α, and IL-18 also inhibit pro-inflammatory cytokines, highlighting the complex interplay and balance within the immune system to maintain health and respond to disease [1].

### Interleukin-10 (IL-10)

IL-10 is a potent anti-inflammatory cytokine that is notable for its ability to suppress the production of inflammatory cytokines, such as TNF-α, IL-6, and IL-1, by activated macrophages. IL-10 can also increase the levels of endogenous anti-cytokines and decrease receptors for pro-inflammatory cytokines, thereby effectively counteracting the production and function of pro-inflammatory cytokines at multiple levels. IL-10 is secreted by T and B cells, monocytes, macrophages, and epithelial cells [62]. Studies have demonstrated that acute administration of IL-10 can inhibit the onset of spinally mediated pain enhancement in various animal models, including peripheral neuritis, spinal cord excitotoxic injury, and peripheral nerve injury [63,64,65]. Conversely, blocking spinal IL-10 has been shown to prevent and even reverse established neuropathic pain behaviors [63,64,65]. IL-10 has the potential to interfere with neutrophil function, which plays an essential role in moderating systemic inflammation [62]. Clinical investigations suggest that low levels of IL-10, in addition to the anti-inflammatory cytokine IL-4, may play a crucial role in chronic pain [66]. These findings suggest that IL-10 has significant potential as a therapeutic agent for the management of inflammatory and neuropathic pain [67,68,69,70,71,72].

It has been reported that after trauma and major surgery, the plasma levels of IL-10 increase. Studies have shown a relationship between IL-10 release and reduced HLA-DR expression during surgery and in critical illness. There is also a positive correlation between IL-10 levels and increased injury severity [73,74,75,76]. However, other researchers have found that IL-10 secretion remains unchanged in patients undergoing major surgery [77,78], and is even decreased in trauma patients [79]. In fracture soft tissue hematomas, IL-10 was present in high concentrations during the first 24 h post-injury, while IL-10 was rarely detectable in plasma from these patients [80]. Therefore, the use of IL-10 as a parameter for monitoring trauma severity may be questionable.

IL-10 is a crucial anti-inflammatory cytokine that suppresses pro-inflammatory cytokine synthesis both in vivo and in vitro [76,81]. Clementsen T. et al. [82] demonstrated only modest changes in systemic and local values of IL-10. Both the pro-inflammatory cytokine TNF-α and anti-inflammatory cytokine IL-10 do not seem to play any role in stable surgical patients; they appear predominantly in unstable patients who develop symptoms of circulation failure or sepsis. Their study indicated that total hip replacement surgery in otherwise healthy patients is associated with reasonably modest reactions to inflammation, both systematically and locally [82].

## 5. Pain Therapy Using IL-10 Gene Therapy

Research has suggested a strong correlation between IL-10 dysregulation and chronic pathological pain. Studies have shown that IL-10 is upregulated following peripheral nerve injury, potentially as a compensatory mechanism to restore homeostasis. However, as pathological pain intensifies, IL-10 levels diminish below baseline in critical pain-related anatomical areas [83,84,85,86,87]. Although direct application of IL-10 can provide immediate pain relief, its effects are transient and may necessitate repeated injections [88]. Gene therapy has emerged as a promising method to maintain stable IL-10 levels, particularly in conditions requiring localized expression, such as low back radiculopathy. Initial attempts employed viral vectors for lumbar spinal delivery but encountered limitations due to short-lived effects and potential immune responses against viral components [66,88,89,90,91,92,93]. Subsequently, non-viral approaches to IL-10 gene therapy have been shown to be remarkably effective despite their general inefficiency in gene transfer. These methods have consistently provided profound and enduring pain relief in various animal models through increased peri-spinal IL-10 production and decreased levels of pro-inflammatory mediators. In particular, non-viral IL-10 gene therapy in neuropathic animals alleviates pain and mediates anti-inflammatory responses in the dorsal root ganglia (DRG) in the absence of endogenous IL-10 [94,95,96,97,98,99,100,101,102,103,104]. This therapy leads to an increase in anti-inflammatory transforming growth factor-beta 1 (TGF-β1) and a reduction in pro-inflammatory TNF-α mRNA levels, indicating that alterations in cytokine profiles within the DRG can effectively mitigate spinal neuropathic pain mechanisms after peripheral nerve damage [104]. Furthermore, this approach demonstrated that non-viral gene therapy may be a viable alternative to viral methods, providing a sustained therapeutic effect without complications associated with viral vectors. To date, there have been no reports of sex differences in the efficacy of IL-10 treatment for chronic pathological pain [104].

## 6. Osteoarthritis Biomarkers

Osteoarthritis (OA) is a complex, multifactorial process that leads to cartilage remodeling and structural and functional changes [105]. Osteoarthritis is one of the most common causes of disability among the elderly, and can affect every joint in the human body, being most prevalent in the hip and knee. OA is a chronic condition that causes joint pain, inflammation, and loss of the articular cartilage [106]. Risk factors for OA can be divided into person-level factors (age, gender, obesity, genetics, and diet), joint-level factors (injury, malalignment and abnormal loading of the joints) and occupational factors (knee bending, heavy lifting, and squatting), that interact in a complex manner [107,108,109,110]. Other authors present similar results based on data from systematic reviews and meta-analysis; their results suggest that lifestyle-related risk factors in the form of BMI, serum calcium, and LDL have true biological effects on the development of OA [111]. Early diagnosis of cartilage lesions is essential for fast and accurate treatment. Available diagnostic methods include conventional X-ray, ultrasound, and magnetic resonance imaging. Unfortunately, these diagnostic modalities are not suitable for large screening purposes. Vibroarthrography has been proposed in the literature by different authors as a screening method for cartilage lesions [106,112]. Different treatment options for early knee arthritis have been proposed. Arthroscopic microfracture might be an option in the early stages of osteoarthritis, and improves function and also reduces pain in older groups of patients [113].

Total joint arthroplasty (TJA) is a common treatment for OA at the end stages, but it can cause postoperative inflammation, which directly affects the levels of cartilage degradation biomarkers, such as proteoglycan-4 (PRG4) and matrix metalloproteinase-9 (MMP-9) [105,114,115]. PRG4 is a glycoprotein that protects joints and is decreased in individuals with OA, whereas MMP-9 contributes to articular cartilage loss and is elevated in patients with OA [105,114,115]. Pro-inflammatory markers such as IL-1, IL-6, and CRP upregulate MMP-9 and initiate the inflammation cascade, leading to cartilage degradation [105,114,115].

Many pro-inflammatory biomarkers have been implicated in the pathophysiology of OA or other inflammatory processes, such as IL-1, IL-2, IL-6, IL-15, IL-18, CRP, leptin, and TNF-α [105,116,117,118,119,120,121,122,123,124,125]. IL-6 is known to acutely increase postoperatively after TJA and is correlated with the severity of pain in patients with OA. CRP is an acute-phase reactant that is positively correlated with OA joint pain and has been shown to predict operative and postoperative outcomes [105,116,117,126,127,128,129,130].

Studies have identified PRG4 as a proteoglycan that facilitates lubrication and mobility in joints, and the inflammatory process and matrix-degrading enzymes result in a decrease in its synthesis [105,115,131,132,133,134,135,136,137,138].

Mitochondrial dysfunction, along with an impaired ability to neutralize reactive oxygen species (ROS), leads to sustained production of pro-inflammatory cytokines like interleukin-1 beta (IL-1β) and interleukin-6 (IL-6). This chronic inflammatory state hinders the shift of macrophages from a proinflammatory to an anti-inflammatory phenotype, a process that is crucial for the resolution of inflammation. Since repolarization is essential for controlling inflammatory responses in various tissues, its impairment can significantly impact the onset and progression of osteoarthritis, potentially exacerbating the pathology of the disease [139,140,141,142,143,144,145,146].

Some studies have highlighted a connection between obesity, inflammatory processes, and osteoarthritis, particularly focusing on the roles of macrophages and inflammatory mediators [147]. In rat [147] and rabbit [148] models of diet-induced obesity and surgically induced OA, an increase in pro-inflammatory macrophages and inflammatory mediators such as interleukin-1 beta (IL-1β), interleukin-6 (IL-6), and tumor necrosis factor (TNF) has been observed within the synovium, contributing to the progression of OA [148]. Additionally, clinical observations suggest that obesity in patients with OA is correlated with increased pain [149,150,151,152,153,154,155,156,157,158]. Fibroblast-like synoviocytes (FLS) extracted from obese patients with hip OA were found to secrete higher levels of IL-6 compared to FLS from lean patients, particularly when interacting with chondrocytes through the adipokine leptin. This indicates that not only does obesity exacerbate inflammatory processes, but also that the interaction between different cell types in the joint can further enhance inflammation [121,159,160,161]. Despite the precise relationship between obesity and synovial inflammation, as evidenced by conventional MRI findings, improvements in knee pain following significant weight loss were not attributed to reductions in synovitis or bone marrow lesions (BMLs), as assessed by MRI. Instead, pain relief was partially attributable to increased pressure-pain thresholds and reduced depression scores. Moreover, weight loss did not seem to lead to improvements in MRI-detected BMLs or synovitis scores, corroborating the findings of earlier studies [162,163,164].

These findings suggest that while obesity is linked to increased inflammation and pain in OA, weight loss may alleviate symptoms through mechanisms other than the direct reduction of joint inflammation, as observed by MRI. The complexity of OA in the context of obesity is underlined by the multifaceted nature of pain and inflammation as well as the potential role of other factors such as mechanical stress, depression, and systemic inflammation.

In summary, there was an increase in inflammation and matrix metalloproteinase-9 (MMP-9) activity coupled with a reduction in proteoglycan 4 (PRG4). Diminished levels of PRG4, known for its protective role in joint lubrication, render the joint more susceptible to damage. The increase in MMP-9 activity exacerbates the breakdown of the extracellular matrix (ECM) of articular cartilage and further processes osteopontin (OPN), enhancing the inflammatory feedback loop and perpetuating the degradation cycle. Additionally, elevated thrombin levels in patients with OA further activate the inflammatory cascade, augment MMP-9, and process OPN, intensifying joint deterioration [105,115,116,117,165,166]. PRG4 plays a pivotal role in mitigating inflammatory responses and modulating the activity of matrix-degrading enzymes such as MMP-9, which are critical in the progression of OA. Equilibrium among PRG4 levels, inflammatory mediators, and degrading enzymes determines the pathogenesis of OA. Increased inflammatory activity may suppress PRG4 production in chondrocytes by activating inflammasomes and other regulatory mechanisms. Conversely, increased PRG4 levels influence the production of inflammatory biomarkers in OA. This delicate balance, influenced by various predisposing factors, varies significantly among patients [105,114,115,116,117,165,166]. 

## 7. Orthopedic Surgeries and Inflammatory Signatures

Orthopedic surgical procedures such as total hip and knee arthroplasties, surgeries that alleviate pain, improve the quality of life of the patients, and restore joint functions can lead to changes in a patient’s hemodynamic, metabolic, and immune responses during the postoperative period [167,168]. Osteoarthritis (OA) is the primary cause of persistent musculoskeletal pain and invalidity [169,170]. 

A local inflammatory response in the surgical wound helps limit tissue damage and promote the healing process. This reaction is characterized by the production of various pro-inflammatory mediators, including interleukin (IL)-1 and tumor necrosis factor α (TNF-α), which can induce the release of other cytokines such as IL-6 and lead to postoperative complications after joint replacement [171]. Hip joint osteoarthritis is known to be associated with chronic pain, including the synthesis of pro-inflammatory cytokines such as IL-1, IL-6, and IL-8, and growth factors that play a definitive role in the inflammatory response pathophysiology. 

However, some studies have shown that surgery can also induce immunosuppression. For example, phagocytic activity in specific cells (PMN, monocytes, and macrophages) is reduced during the postoperative period, which makes patients more susceptible to infection [172,173,174,175,176,177,178,179,180,181,182,183,184]. 

Immature monocytes and granulocytes, called myeloid-derived suppressor cells (MDSCs), are recruited to tissues during prosthetic joint infection (PJI) where they exert anti-inflammatory effects [174,175,177,185,186,187].

An increased level of anti-inflammatory molecules, including IL-4, IL-10, soluble tumor necrosis factor receptor 1 (sTNFR1), IL-1 receptor antagonist (IL-1Ra), and transforming growth factor β (TGF-β) can also be produced during the postoperative period following total joint arthroplasties [188].

When analyzing routine clinical markers to evaluate the magnitude of the systemic inflammatory response after elective surgeries, IL-6 and C-reactive protein seem to be among the most commonly used markers. It is known that levels of IL-6 are most elevated approximately 18–24 h after surgery (but have been reported to increase from around 2 h after surgery), and CRP levels peak at approximately 48 to 72 h (but the elevation of this marker starts at 4–6 h after operation) [189].

The initial stage of pain treatment in osteoarthritis involves the use of non-steroidal anti-inflammatory drugs (NSAIDs) and acetaminophen (paracetamol) [190]. In some cases, opioid analgesics or intra-articular glucocorticoids can be prescribed. Patients with more severe OA stages may require surgical treatment [190]. After total hip replacement surgery, regular analgesic treatments include opioids, acetaminophen, metamizole, and NSAIDs. When used in combination, these drugs produce better pain relief owing to their synergistic effects [191]. Despite advancements in pain relief medication, many patients still do not receive effective therapies. Genetic factors are key contributors to interpatient variability in pain threshold and pain management [17]. 

It is also known that implant-derived wear particles are involved in activating pro-inflammatory mediators and mononuclear precursor cells and are associated with the macrophage response [192]. Kaufman A.M. et al. reported, based on the results of their in vitro study, that Ti-6Al-4V particles are the most stimulatory for cytokine expression, CoCr and alumina as being mildly stimulatory, and UHMWPE particles have the least stimulatory effect [193]. In the late 1990s, a French study suggested that interleukin-6 (IL-6) serum levels could be a significant biomarker for detecting osteolysis in patients who have undergone total hip arthroplasty. The study categorized patients into groups based on clinical and radiographic evidence, highlighting the potential of IL-6 as an indicator of both active and preclinical osteolysis. These findings suggest a complex interplay between IL-6 levels, prosthetic wear, bone mineral density changes, and manifestation of osteolysis [194].

## 8. Fascia Iliac Compartment Block (FICB)—Serum NLRP3 and the Role of Inflammatory Biomarkers in the Field of Orthopedic Surgeries

In addition to the use of analgesics and nonsteroidal anti-inflammatory drugs in the management of pain associated with surgeries, the application of fascia iliac compartment block (FICB) has been reported in several surgeries to obtain good results, such as total hip arthroplasty and fracture management [195,196,197,198,199]. The activation of inflammation-related factors during pain is associated with the development of pain. Postoperative pain and inflammatory biomarkers, as well as the nucleotide-binding domain and leucine-rich repeat (NLR) family, particularly pyrin domain-containing 3 (NLRP3), play a significant role in the inflammatory response and pain management after surgery for such fractures [50,200,201,202,203]. The NLRP3 inflammasome is a key component of the innate immune system that regulates the production of pro-inflammatory cytokines, such as IL-1β, which are vital in the inflammatory process. Managing these inflammatory factors can be critical for controlling postoperative pain and enhancing healing [50,199,200,201,202,203].

## 9. Plasminogen Activator Inhibitor-1 and Its Role

Plasminogen activator inhibitor-1 (PAI-1), a member of the serine protease inhibitor (serpin) family, plays a crucial role in regulating the plasminogen/plasmin system. Since its identification, the physiological and pathophysiological significance of PAI-1 has been thoroughly investigated in human and animal disease models. Research has established associations between PAI-1 and various conditions such as cardiovascular disease (CVD), metabolic syndromes, aging, cancer, tissue fibrosis, inflammation, and neurodegenerative disorders. Consequently, research on PAI-1 inhibitors has been pursued to delve deeper into PAI-1’s role in these diseases and to assess their therapeutic potential [204,205].

PAI-1, a single-chain glycoprotein consisting of 379 or 381 amino acids, is the main inhibitor activator of plasminogen, tPA and uPA, which are key components of the fibrinolytic system [206]. PAI-1 is synthesized and released by various cell types in different tissues, and its expression and release are tightly regulated by several factors, including growth factors, hormones, inflammatory cytokines, glucose, and endotoxins [204,207]. Although plasma PAI-1 circulates at relatively low levels (5–50 ng/mL), it is predominantly in the active conformation. In contrast, platelets retain the main pool of PAI-1 (up to approximately 300 ng/mL), with only 2–5% being functionally active upon platelet lysis [208,209]. However, recent studies have suggested that platelet activation can upregulate the synthesis of PAI-1 through translationally active PAI-1 messenger RNA, resulting in the release of PAI-1 and partial retention of PAI-1 on platelets in its active conformation [210]. PAI-1 contains three potential glycosylation sites, and its glycosylation pattern is tissue-type-specific, with Asn209 and Asn265 being the glycosylation sites [210].

Acute phase proteins, such as PAI-1, are crucial in the body’s inflammatory and immune responses to both infectious and non-infectious injuries [205]. Conditions such as obesity, type 2 diabetes, and metabolic syndrome frequently coexist with a chronic inflammatory state marked by elevated production of inflammatory adipokines, including interleukin-6 (IL-6) and tumor necrosis factor-alpha (TNF-α) [211]. These factors are known to stimulate the expression of PAI-1 in adipose tissue [212]. The resultant heightened levels of PAI-1 exacerbate inflammation in the adipose tissue by promoting the infiltration of inflammatory macrophages. In addition to the established positive relationship between inflammatory indicators and PAI-1 concentrations, a connection has also been identified between PAI-1 and the regulation of lipid metabolism in the context of obesity [205,212].

In Table 1, a summary of the main cytokines is shown, their properties highlighted, and properties that are dependent on the microenvironment are specified. Most of them have dual effects according to their microenvironment [213].

## 10. Results and Discussion

Pro- and anti-inflammatory cytokines help regulate the immune system. They maintain a balance between potential injury and excessive inflammation under normal conditions. However, under pathological conditions, the balance between pro- and anti-inflammatory cytokines can be disrupted, leading to either excessive immune response or immune suppression. This imbalance can result in organ dysfunction, immunity, and infection, as well as wound healing and pain after surgery. In musculoskeletal trauma, such as during surgery, the body’s response is an initial elevation of pro-inflammatory cytokines in the blood, followed by later activation of anti-inflammatory cytokines to restore balance. Osteoarthritis of the hip joint is characterized by an increase in pro-inflammatory mediators, particularly IL-6, IL-8, and TNF-α, in the synovial fluid. Non-steroidal anti-inflammatory drugs (NSAIDs) are commonly used to treat osteoarthritis and have been shown to decrease IL-6, TNF-α, and vascular endothelial growth factor (VEGF) levels in the synovial fluid, improving joint pain and function [226,227].

Total hip and knee replacement surgery involves significant trauma to both the soft and bony tissues, which can lead to intense pain. It is necessary to note that the amount of tissue damage incurred during surgery can be directly responsible for the increase in inflammation in the perisurgical region. After tissue injury, pro-inflammatory cytokines such as IL-6 are overproduced. The increase in IL-6 concentrations triggers the synthesis of acute-phase proteins, such as C-reactive protein (CRP), as well as activation of the innate immune system [189,228]. Bialecka M. et al. report in their study of patients that undergo total hip replacements that individuals carrying the IL-6 –174G allele, which is associated with increased gene expression, are more likely to have higher levels of IL-6, leading to increased inflammation and greater stimulation of nociceptors by inflammatory mediators [229]. Their results support this hypothesis, as patients who carried the G allele required higher doses of analgesics, specifically opioids, in the early postoperative period than those who did not carry the G allele. Notably, these findings contradict the results of Reyes’ study published in 2008 [230], which did not find a significant correlation between the IL-6 –174G allele and analgesic requirements after total knee replacement surgery. Patients with the IL-6 –174GG genotype have been found to have higher levels of IL-6 than CC carriers. This results in an increased demand for opioids within 24 h of surgery [229]. Although morphine doses and pain intensity gradually decreased during the postoperative period, a subset of patients still experienced significant pain in the study by Bialecka et al. [229]. Patients with at least one IL-6 –174G allele (GG homozygote and GC heterozygote) required opioids significantly more often, even on days 3 and 4, than CC homozygous subjects [229].

One of the earliest studies performed on patients who underwent total hip arthroplasties in France that evaluated the blood concentrations of different cytokines was published by Hernigou et al. [194]. The authors performed a screening for the presence of circulating cytokines IL-1β, IL-6, IL-8, and TNF-α. The study was performed on patients that had a total hip arthroplasty and were free of inflammatory diseases and infections. The patients were included in one of four groups: Group A—follow-up of more than 10 years who had evident osteolysis; Group B, with a follow-up of >10 years and no osteolysis; Group C, patients with a shorter follow-up than Group A; and Group D, control patients. In the control groups (C and D), the levels of cytokines, including IL-6, IL-1β, TNF-α, and IL-8, were undetectable and fell below the defined normal limits (IL-6 < 15 pg/mL, IL-1β < 50 pg/mL, TNF-α < 20 pg/mL, and IL-8 < 8 pg/mL). Additionally, the CRP levels in these groups were within the normal range (<5 mg/L). Group A patients were characterized by detectable elevations in IL-6 and CRP across all patients (IL-6 ranged from 30 to 72 pg/mL; CRP ranged from 6 to 12 mg/L), and exhibited significantly higher average levels of IL-6 (41 pg/mL, standard deviation 17 pg/mL) and CRP (10 mg/L, standard deviation 2.4 mg/L) compared to the controls; these differences were statistically significant (*p* = 0.002 for IL-6). In Group B, elevated serum levels of IL-6 and CRP were noted in 60% of patients. The mean IL-6 level in this group was 22 pg/mL (standard deviation 6.7 pg/mL), and the mean CRP level was 7.7 mg/L (standard deviation 2.8 mg/L), with these increases being statistically significant (*p* = 0.02) when compared with Group C. Furthermore, a comparison between Groups A and B revealed a significantly higher increase in IL-6 levels in Group A (*p* = 0.002), indicating a more pronounced inflammatory response in this group. This study underscores the variability in inflammatory marker levels among patients with different conditions and highlights the importance of these biomarkers for evaluating and understanding the inflammatory process. The use of threshold values for undetectable levels in statistical analysis provides methodological insight into handling such data, with the observed differences in cytokine and CRP levels offering a basis for further investigation into their clinical significance [194].

Watt D. et al. report, based on their literature review, that the time of peak response for IL-6 after elective surgeries is between 12 and 24 h, and that the magnitude of the serum concentration is associated with the operative injury and the invasiveness of the surgical procedure. In terms of elective surgeries in the field of orthopedics, primary total knee arthroplasties show peaks between 6 and 24 h [189]. Similar results were reported in other studies [231]. In terms of the CRP response, peak levels were observed 24–72 h after surgery. Peak levels of CRP concentrations in the field of both primary and revision total hip arthroplasties are encountered 24–72 h after surgery. The authors concluded that both IL-6 and CRP seem to be valuable markers for the assessment of systemic inflammatory response after elective surgeries, such as those in the orthopedic field [189].

Zhu K. et al. performed a randomized control study to investigate the role of FICB on postoperative pain and inflammatory factors including NLRP3 in patients with femoral intertrochanteric fractures [199]. The dynamic alterations and association between NLRP3 and inflammatory markers were analyzed in both cohorts of patients. The temporal progression of serum NLRP3 levels and its association with inflammatory markers were also evaluated in this study. From 1 h to 24 h postoperatively, there was a significant elevation in the concentrations of NLRP3, CRP, IL-6, and TNF-α relative to baseline values, followed by a subsequent decline. Notably, the levels of NLRP3 and IL-6 were markedly reduced in patients receiving FICB at time points of 1, 6, 24, 48, and 72 h post-surgery in comparison to the control cohort (*p* < 0.05). Furthermore, TNF-α levels were significantly decreased in the FICB group of enrolled patients at 1 h and 6 h post-operation, while CRP levels were significantly lower at 1 h and 24 h post-operation than in the control group (*p* < 0.05). No notable differences were observed at one week post-surgery. Additionally, a significant positive correlation was identified between NLRP3 and IL-6 levels 1 h post-surgery (*p* < 0.05). A positive correlation was also observed between CRP levels and Visual Analog Scale (VAS) scores 1 h post-surgery (*p* < 0.05). Conversely, no significant correlations were detected between VAS scores and other inflammatory markers, including CRP, IL-6, and TNF-α [199].

Similar results were also published by Chen et al., who enrolled in their study 34 patients who underwent total hip replacement [232]. Before surgery, IL-6 levels were comparable across the study groups, with a peak observed 24 h postoperatively for each group before a subsequent decline. Similarly, the preoperative CRP levels did not differ significantly between the groups. Post-surgery, CRP levels surged and peaked on the second day. Preoperative CK levels were similar across the groups, increased after surgery, and peaked at 24 h, mirroring the IL-6 trend. Significant differences were noted in IL-6, CRP, and CK levels at various time points (*p* < 0.001 for each). However, when comparing the areas under the curve (AUCs) for IL-6, CRP, and CK across different groups, no significant differences were found (*p* = 0.23 for IL-6, *p* = 0.637 for CRP, and *p* = 0.448 for CK) [232].

The fascia iliac compartment block (FICB) has been shown to reduce postoperative pain, particularly in femoral neck fractures and total hip arthroplasty (THA) [233]. Some studies have suggested that FICB may also have a positive effect on postoperative cognitive status [234,235]. In a recent study, it was found that FICB can reduce postoperative pain after femoral intertrochanteric fracture, but it did not seem to affect the recovery of hip function [235]. Research shows that inflammation is closely related to pain. After THA, levels of pro-inflammatory factors such as CRP and IL-6 increase and begin to decrease three days after surgery [236]. The release of these cytokines can be reduced using local infiltration analgesia. Studies have also found that synovial fluid PGE2, IL-6, IL-8, and TNF-α levels increase after THA and are correlated with pain levels during walking. Postoperative CRP and IL-6 levels have been found to be higher in patients with postoperative cognitive dysfunction after THA. NLRP3 is a newly discovered factor associated with the progression of many diseases and may be related to inflammation [236]. Feng T. et al. assessed the role of FICB and postoperative cognitive dysfunction and serum inflammatory cytokines in a recent published study on patients that underwent total hip replacement surgery [237]. All enrolled patients underwent general anesthesia with endotracheal intubation and an FICB that was performed with an ultrasound-guided inside approach (Inside group) or outside approach (Outside group). The authors found that the Outside group had a higher ratio of re-fixing the catheter, longer intubation time, and greater use of ropivacaine dosage at 48 h after surgery than the Inside group (*p* < 0.05). However, the depth of cannulation in the Outside group was lower than that in the Inside group (*p* < 0.05). The VAS scores were similar between the Inside and Outside groups, except at 24 h after surgery. The use of patient-controlled analgesia from 24 to 48 h after surgery in the Outside group was significantly higher than that in the Inside group (*p* < 0.05). The Outside group had higher Mini-Mental State Examination scores and a higher incidence of postoperative cognitive dysfunction than the Inside group did. Additionally, serum IL-1β levels that were determined at 1 and 6 h after surgery and serum IL-6 levels at 1, 6, 24, and 48 h after surgery in the Outside group of patients were significantly higher than those in the Inside group of enrolled patients (*p* < 0.05). Other studies have also demonstrated that elevated serum inflammatory cytokines in patients who undergo total hip arthroplasties, mainly IL-1β and IL-6, are associated with postoperative cognitive dysfunction [238,239].

Kuchálik J. et al. performed a sub-analysis of a larger double-blind study involving 56 patients who underwent total hip arthroplasty under spinal anesthesia. The femoral nerve block (FNB) group received an ultrasound-guided femoral nerve block with 30 mL of 7.5 mg/mL ropivacaine (225 mg) and saline (151.5 mL of saline peri-articular during surgery). The local infiltration analgesia (LIA) group was administered 30 mL saline for femoral nerve block and a peri-articular mixture of ropivacaine 2 mg/mL (300 mg, 150 mL), ketorolac 30 mg (1 mL), and adrenaline 0.5 mg (0.5 mL). After 23 h, an additional 22 mL of the LIA mixture was injected peri-articularly in the LIA group, and 22 mL of saline was administered in the FNB group through a catheter. A comprehensive assessment of pro- and anti-inflammatory cytokines was performed by the authors preoperatively and at 4 h and 3 days postoperatively, using a commercial kit. CRP levels, platelet counts, and white blood cell counts were measured before and after surgery. Postoperatively, the authors reported an overall increase in pro-inflammatory cytokines, which normalized after three days. IL-6 levels were notably lower in the LIA group than in the FNB group 4 h after surgery (*p* = 0.015). No significant differences in the levels of other cytokines were observed between the groups. CRP levels were significantly higher in the FNB group than in the LIA group three days post-surgery (*p* < 0.001), with no other significant differences noted. The authors also reported that patients receiving LIA had a significantly lower concentration of plasma IL-6 4 h after surgery. The authors concluded that local infiltration analgesia demonstrated a limited yet transient effect on reducing postoperative inflammation in patients undergoing total hip arthroplasties, potentially attributed to the local application of ketorolac and/or local anesthetics in the LIA mixture [227].

Liu X. et al. [240] performed a study that aimed to evaluate the effectiveness of combining fascia iliac compartment block (FICB) with dexmedetomidine (DEX) for postoperative pain and inflammation management in elderly patients following total hip arthroplasty. A total of 119 elderly patients were randomized into three groups from March 2016 to December 2018: a control group receiving routine general anesthesia, an FICB group receiving FICB post-surgery, and a combined group receiving both DEX pre-treatment and FICB post-treatment. The outcomes were assessed by the authors through measurements of serum interleukin-1β (IL-1β), IL-6, and C-reactive protein (CRP) levels using ELISA, pain intensity using the visual analog scale (VAS) at 12, 24, 48, and 72 h post-surgery, and patient-controlled intravenous analgesia (PCIA) usage within 48 h post-surgery. Sleep quality was evaluated using the Pittsburgh Sleep Quality Index (PSQI) before and one month after surgery. The results indicated that VAS scores were significantly lower in the combined group than in both the control and FICB groups at all time points measured. The combined group demonstrated notably shorter PCIA pressing times. Serum levels of IL-1β, IL-6, and CRP were lowest in the combined group, suggesting reduced inflammation. Additionally, the PSQI scores improved significantly in the combined group compared to the other groups, indicating better postoperative sleep quality. No severe side effects or significant differences in the side effects were observed across the groups. The study concluded that the combination of FICB and DEX significantly reduced postoperative pain, decreased inflammatory markers, and improved sleep quality in elderly patients undergoing total hip arthroplasty [240].

A recently published study [237] aimed to compare the anesthetic effects of the inside versus outside approaches of FICB in THA patients, particularly focusing on postoperative cognitive dysfunction (POCD) and serum inflammatory cytokine levels. A total of 60 patients undergoing THA from January 2021 to December 2021 were divided into two groups based on the FICB approach: the Inside group and the Outside group. The study assessed several primary outcomes, including the use of ropivacaine, pain levels via the visual analog scale (VAS), use of patient-controlled analgesia (PCA), mini-mental state examination (MMSE) scores, incidence of POCD, and serum levels of IL-1 and IL-6. The secondary outcomes evaluated were surgical indicators and quality of anesthesia cannula placement. The results indicated that the Outside group had higher ropivacaine usage, longer intubation times, and a higher incidence of re-fixing the catheter compared to the Inside group, which also exhibited deeper cannulation (all *p* < 0.05). While VAS scores were similar between the groups, PCA usage was significantly higher in the Outside group between 24 and 48 h post-surgery. Furthermore, MMSE scores and incidence of POCD were higher in the Outside group. Serum levels of IL-1β at 1 and 6 h post-surgery and IL-6 levels at 1, 6, 24, and 48 h were significantly higher in the Outside group than in the Inside group. The study concluded that the inside approach of the FICB demonstrated better anesthetic effectiveness, improved postoperative analgesia, reduced need for postoperative analgesics, lower incidence of POCD, and decreased serum cytokine levels in patients undergoing THA than the outside approach [237].

Therefore, the local infiltration of ketorolac and local anesthetics may reduce pain intensity by exerting local anti-inflammatory effects. This partly explains the known analgesic effect of local infiltration analgesia (LIA) [199,226,227,241,242,243,244,245].

Kwak S. et al. report the results of an observational study in which the authors enrolled 30 patients, 15 of whom underwent minimally invasive total hip arthroplasty [62]. A visual analog pain scale was used to record pain levels. Blood tests were conducted to determine the extent of muscle injury and systemic inflammatory response. These tests included measuring creatinine kinase (CK) and aldolase enzymes, as well as the pro-inflammatory cytokines interleukin (IL)-6 and IL-8, and the anti-inflammatory cytokines IL-10 and IL-1 receptor antagonists (ra). All evaluations were performed simultaneously at 7:00 AM, prior to the intervention [62]. During the study, it was found that the group receiving minimally invasive total hip arthroplasty showed significantly lower levels of serum creatinine kinase, IL-6, IL-10, and IL-1ra on postoperative days 1 and 3, compared to the control group. In addition, the levels of IL-8 were also found to be significantly lower on day 7 after surgery. Before the surgery, the IL-6 level was measured to be 13.9 ± 0.3 pg/mL in the study group and 14.2 ± 0.7 pg/mL in the control group, with no significant difference between them. However, on postoperative day 1, the serum IL-6 concentration increased to 66.8 ± 2.1 pg/mL in the study group and 176.3 ± 2.5 pg/mL in the control group. On postoperative day 3, these values were 42.9 ± 1.1 pg/mL and 105.1 ± 1.8 pg/mL, respectively, with a significant difference (*p* = 0.0, *p* = 0.0). However, this significant difference disappeared on postoperative day 7. On postoperative day 14, the levels of IL-6 were found to be similar in both groups. Before surgery, the serum IL-10 level was not significantly different between the two groups, with 3.2 ± 1.1 pg/mL in the study group and 3.3 ± 0.9 pg/mL in the control group. On postoperative day 1, the IL-10 level increased in both groups, but the level in the study group (12.2 ± 1.9 pg/mL) was significantly lower than that in the control group (21.7 ± 2.8 pg/mL; *p* = 0.0). On postoperative day 3, it had decreased slightly in both groups (10.6 ± 1.8 pg/mL in the study group and 14.6 ± 2.0 pg/mL in the control group; *p* = 0.0). However, this difference disappeared by postoperative day 7. The concentration of serum IL-1ra was also not found to be significantly different between the groups before surgery, with 235.4 ± 8.9 pg/mL in the study group and 245.1 ± 7.7 pg/mL in the control group. On postoperative day 1, it increased to 695.0 ± 10.2 pg/mL in the study group and 1370.8 ± 16.7 pg/mL in the control group (*p* = 0.0). On postoperative day 3, it had decreased in both groups (543.6 ± 10.1 pg/mL in the study group and 938.3 ± 15.9 pg/mL in the control group; *p* = 0.0). These results indicate that minimally invasive total hip arthroplasty could produce a lesser inflammatory reaction than the classical approach in the early recovery stage after surgery.

Bergin et al. [246] investigated the extent of muscle damage resulting from anterior and posterior approaches during minimally invasive total hip arthroplasty. The study included 29 patients who underwent the procedure through a DDA (direct anterior approach) and 28 patients who underwent the same procedure through a posterior approach. The authors measured serum creatine kinase (CK), CRP, IL-6, IL-1β, and TNF-α levels preoperatively, and on postoperative days 1 and 2. The results indicated that the levels of inflammatory markers were slightly lower in the direct anterior approach group than in the posterior approach group. The study found that the increase in CK levels in the posterior-approach group was 5.5 times higher than that in the anterior-approach group in the post-anesthesia care unit and nearly twice as high cumulatively. The authors concluded that the DDA total hip arthroplasty approach used in this study caused significantly less muscle damage than the posterior surgical approach, as indicated by serum CK levels. However, additional clinical studies are needed to delineate the clinical importance of increased CK levels. The results of the measurements of the inflammation biomarker levels suggest that the overall physiological burden is comparable between the anterior and posterior approaches [246].

Montaghedi et al. [247] investigated in their study the effect of obesity on inflammation and pain after total hip arthroplasty. Although obesity is known to cause chronic low-grade inflammatory response, its effect on postoperative outcomes remains unclear. This study aimed to determine the association between obesity and the severity of postoperative inflammatory response, as measured by circulating cytokine levels, blood cell reactivity, and postoperative pain, evaluated by verbal pain scores and analgesic consumption. In their prospective cross-sectional study of 60 patients, the enrolled patients were divided into normal-weight, overweight, and obese groups. Blood samples were collected before and 24 h post-THA to assess C-reactive protein and cytokines such as IL-1β, IL-2, IL-6, IL-8, and TNF-α. Pain was measured using verbal scores and by monitoring analgesic use during the first 24 h post-surgery. The results showed no correlation between the body mass index (BMI) and spontaneous levels of circulating cytokines postoperatively. However, upon ex vivo activation of blood leukocytes, a significant positive correlation was observed between BMI and levels of IL-1β, IL-6, and TNF-α, suggesting that obesity could prime the immune system for an exaggerated postoperative inflammatory response. Interestingly, obesity was not linked to increased postoperative pain or analgesic use [247]. In conclusion, this study found that obesity is associated with an enhanced pro-inflammatory state post-THA, as indicated by increased cytokine reactivity. This suggests that further large-scale studies are needed to explore the specific effects of obesity on surgical outcomes including pain. This could lead to better postoperative management strategies for obese patients undergoing THA.

Proinflammatory cytokines can also be used to evaluate the relationship between inflammatory responses and complication rates after total hip arthroplasties (THA). Few studies have explored the relationship between inflammatory response and postoperative complication rates. One of these studies [239] aimed to investigate the early inflammatory response during the first three days after THA and to identify the relationship between the inflammatory response and the estimated complication rate following surgery. This prospective, non-randomized cohort study enrolled 148 patients who underwent unilateral THA. Blood samples were collected preoperatively on the morning of the surgery and at 24, 48, and 72 h after surgery, and C-reactive protein (CRP) and interleukin-6 (IL-6) levels in the peripheral blood were measured. Pre- and intraoperatively, the modified physiological and operative severity scores for the enumeration of morbidity (POSSUM) were recorded. The estimated complication rate was calculated by using this score. The Harris score was used to assess hip function before and after the surgery. IL-6 levels peaked 24 h after surgery, while CRP levels peaked 48 h after surgery, after which both levels declined. The mean Harris score showed a significant increase from 41.62 ± 23.47 before surgery to 72.75 ± 9.13 at 3 days after surgery. However, the Harris score after surgery did not show a significant relationship with either IL-6 or CRP peak levels (*p* = 0.165 and *p* = 0.341, respectively). Both CRP and IL-6 peak levels were significantly and positively correlated with the estimated complication rate following surgery. The estimated complication rate calculated using the POSSUM system was 43 of 148 patients. However, only 28 patients were observed to have postoperative complications during hospitalization, and there was no significant difference between the estimated and observed complication rates (*p* = 0.078). In the group with complications, both CRP and IL-6 peak levels were significantly higher than in the group without complications (both *p* < 0.001). Therefore, the study concluded that there is a significantly positive relationship between the peak levels of both CRP and IL-6 and the estimated complication rate following THA. The inflammatory response can be used to predict the incidence of complications after THA [239].

The influence of age on the regulation of inflammatory response to surgery has been well established, with a positive correlation between aging and increased levels of circulating cytokines. Patient age was the most significant factor contributing to the modulation of the inflammatory reaction in response to surgery. The underlying mechanism for this phenomenon can be attributed to reduced adrenocortical responsiveness to surgical stress, which is often observed in elderly individuals [248]. This response is characterized by decreased production of glucocorticoids by the adrenal glands, which typically inhibit the production of IL-6. Several studies have reported that IL-6 response to surgical trauma can be attenuated by the administration of exogenous glucocorticoids [249]. As such, the attenuated production of glucocorticoids in aged individuals may be responsible for the heightened inflammatory response observed in this population [250].

Minetto M. et al. [251] also investigated the serum interleukin-6 (IL-6) response to total hip replacement. Twenty-one patients received an uncemented total hip prosthesis and venous samples were collected for IL-6 determination before and after surgery. The study found that the IL-6 response was significant, and there was a peculiar heterogeneity of response: the medians of peak levels (82.3 pg/mL) and the areas under the response curve (51.8 pg/mL) distinguished between IL-6 high responders (HR) and IL-6 low responders (LR; *p* < 0.0001). Gender composition was not a significant differentiating factor between the two groups, but HR patients were older than LR patients (*p* < 0.05). The study also found that the increase in IL-6 was correlated with patient age, while its slope was correlated with the duration of the surgical procedure. Furthermore, the HR group had a higher degree of hyperthermia in the days after surgery than the LR group, but there was no evidence of differences in postoperative complications, time to mobilization, or length of hospital stay. The main finding of this study was evidence of significant variability between individuals in IL-6 response to surgery [251].

Reikeras O. et al. [252] also evaluated the changes in the serum pro-inflammatory markers in response to the musculoskeletal surgical trauma. The results showed that the surgical procedure caused a substantial increase in the levels of IL-2R in the serum 6 days after surgery, an increase in levels of IL-6 6 h and 1 d after surgery, an increase in levels of IL-8 6 h after surgery, and an increase in levels of IL-16 after 6 h and 1 d after surgery. In contrast, a significant decrease in serum levels of IL-1Rα was observed at the end of surgery, a decrease in levels of IL-12 at the end of surgery and 6 h after surgery, and a decrease in levels of eotaxin during all phases of the postoperative course [252].

Plasminogen activator inhibitor 1 is a crucial regulator of the fibrinolytic system and is produced by various tissues including the endothelium, liver, and adipose tissue. Its primary role is to inhibit tissue plasminogen activator (tPA), which is responsible for converting the inactive form of plasminogen into plasmin, the active enzyme that breaks down fibrin. Fibrin is a protein that plays a key role in blood clot formation; thus, PAI-1 effectively slows the clot breakdown process. The activity of plasmin is also regulated by α2-antiplasmin, which inactivates plasmin. The balance between plasmin and α2-antiplasmin is an indicator of fibrinolytic activity; a low level of α2-antiplasmin or a high level of plasmin–α2-antiplasmin (PAP) complex signals recent fibrinolysis [253]. Research has demonstrated a correlation between elevated PAI-1 levels and increased body mass index (BMI), suggesting that higher levels of adiposity may contribute to a more inhibited fibrinolytic state. Additionally, high glucose levels have been associated with increased PAI-1 levels, indicating a link between metabolic states and the regulation of fibrinolysis [253]. Burleson A. et al. [253] performed a study to investigate the effect of age, sex, BMI, type of surgery, and tranexamic acid (TXA) treatment on the fibrinolytic system in patients undergoing total joint arthroplasty (TJA). Blood samples from 99 patients with TJA were collected at preoperative clinic appointments and on postoperative day 1. D-dimer, plasminogen activator inhibitor 1 (PAI-1), tissue plasminogen activator (tPA), and antiplasmin activity levels were measured using a commercially available enzyme-linked immunosorbent assay kit. The following data was collected from patient records: age, sex, hemoglobin (Hb) levels, and BMI. The study’s findings indicated that preoperative D-dimer and tPA levels were positively correlated with age, while preoperative antiplasmin was negatively correlated with age. Additionally, body mass index was found to be associated only with preoperative tPA levels. There were no significant differences in postoperative levels of D-dimer, PAI-1, tPA, or antiplasmin between patients who received TXA and those who did not. However, the percentage change in D-dimer and tPA levels was significantly lower in the TXA-treated group compared to the non-treated group. The type of surgery had no impact on the fibrinolytic markers. In summary, this study confirmed that advanced age and elevated BMI play a role in fibrinolytic dysregulation in TJA patients, and TXA appears to decrease fibrinolytic activity [253].

Wasko M. et al. [254] performed a study on 100 participants in which the authors determined the C-reactive protein (CRP), IL-1β, 6 and 8 and NT-proCNP peptide levels before and during the first five postoperative days. C-reactive protein (CRP) levels increased after surgery, peaking on the first day for total knee arthroplasty and on the second and third days for total hip arthroplasty, with significant differences in peak values observed between these groups. Despite a post-peak decrease, the CRP levels remained above the normal range throughout the study period. The TKA group exhibited significantly higher CRP levels on postoperative days 1, 2, 4, and 5, and the overall inflammatory response, as indicated by the area under the curve (AUC), was significantly greater in the TKA group than in the THA group. The dynamics of CRP levels also varied with body mass index. After THA, patients with BMI > 24.9 kg/m^2^ experienced higher CRP levels on all postoperative days, notably from days 2 to 5. However, no significant differences were found among TKA patients, possibly because of the small number of normal-weight patients in this subgroup. IL-6 concentrations peaked on the first postoperative day after TKA and on the second day after THA, with both groups showing a single-phase decline thereafter. The peak IL-6 values differed significantly between the THA and TKA groups. Body weight did not significantly influence the IL-6 levels in either group. For IL-1β and IL-8, no major changes were observed during the first five postoperative days, displaying a sinusoidal pattern starting the day before surgery. Similarly, NT-proCNP levels followed a sinusoidal pattern with no significant difference between the THA and TKA groups. The study discontinued NT-proCNP measurements after the interim analysis of the first 24 patients. Additionally, no statistical differences were noted in CRP, IL-6, and NT-proCNP levels between genders, nor were correlations found with other medical comorbidities or surgical time.

Lubricin, known as proteoglycan 4, serves crucial functions as an anti-adhesive and boundary lubricant in healthy joints, safeguarding against cartilage damage [255]. Its production by synoviocytes and superficial zone chondrocytes ensures its presence in synovial fluid (SF) and articular cartilage surfaces [255]. However, post-injury reductions in SF lubricin levels can precipitate secondary osteoarthritis. Individuals with camptodactyly-arthropathy-coxa vara-pericarditis (CACP) syndrome, characterized by loss-of-function mutations in lubricin, exhibit early onset cartilage failure. This was similarly observed in PRG4 knockout mice, highlighting early cartilage deterioration and chondrocyte changes [255]. Galicia K. et al. [256] performed a study that also acknowledges the role of inflammatory markers like IL-1β and TNF-α in OA pathophysiology, noting their capacity to inhibit lubricin expression and secretion in SF. This study aimed to compare the levels of inflammatory biomarkers and lubricin in patients undergoing total joint arthroplasty and healthy controls to elucidate their roles in OA and to understand the regulatory impact of inflammation on lubricin. Using biochip array technology, various cytokines were profiled in plasma samples collected pre- and post-TJA, and circulating lubricin levels were measured via ELISA. The results of the study revealed that, compared to healthy individuals, TJA patients exhibited significant increases in IL-6, IL-8, VEGF, IL-1β, MCP-1, EGF, and TNF-α, along with notable reductions in lubricin levels both before and after surgery [256]. These observations suggest that inflammatory cytokine surges may trigger mechanisms leading to reduced lubricin levels, thereby elevating OA risk [256].

The levels of pro-inflammatory mediators in unwashed salvaged blood after abdominal aortic aneurysm (AAA) surgery are unknown. To investigate this, we conducted a single-center observational study wherein we compared the levels of pro-inflammatory mediators in the blood salvaged during AAA surgery and total hip replacement surgery. Our study included ten patients scheduled for AAA surgery (Group A) and ten patients for total hip replacement surgery (Group H). Blood samples from the autotransfusion set were obtained during surgery, and arterial samples were obtained before, during, and 6 h after surgery. We determined the interleukin (IL)-1β, IL-6, IL-8, tumor necrosis factor-α, activated complement 3 (C3a), and high-sensitivity C-reactive protein (CRP) levels in the blood samples.

The unknown levels of pro-inflammatory mediators in unwashed salvaged blood from abdominal aortic aneurysm (AAA) surgery were the focus of Lindholm E. et al.’s [257] study. To investigate this, the authors conducted a single-center observational study wherein they compared the levels of pro-inflammatory mediators in blood salvaged during abdominal aortic aneurysm (AAA) surgery and total hip replacement surgery. Their study included ten patients scheduled for AAA surgery (Group A) and ten patients for total hip replacement surgery (Group H). Blood samples from the autotransfusion set were obtained during surgery, and arterial samples were obtained before, during, and 6 h after surgery. The authors determined levels of IL-1β, IL-6, IL-8, TNF-α, activated complement 3 (C3a), and high-sensitivity CRP in the blood samples. The authors found substantially higher levels of IL-1β, IL-6, and IL-8 in salvaged blood in AAA surgery compared to total hip replacement surgery. The levels of pro-inflammatory mediators in unwashed blood from AAA surgery had never been reported before this study [257]. Compared to preoperative values of IL-1β, salvaged blood contained approximately 36-fold higher levels in AAA surgery and approximately 20-fold higher levels in total hip replacement surgery. In the case of IL-6, the authors found approximately 18-fold higher levels in AAA surgery compared to preoperative levels, versus approximately 12-fold higher levels in total hip replacement surgery. The increases in IL-8 in salvaged blood compared to preoperative values were approximately 10-fold higher in AAA surgery versus approximately twofold higher levels in total hip replacement surgery. Additionally, in perioperative arterial blood samples, higher levels of IL-6 were found in AAA surgery than in total hip replacement surgery, and higher levels of IL-1β, IL-6, and IL-8 were found in blood samples after surgery. C3a levels were higher in the total hip replacement surgery group than in the AAA surgery group. Therefore, this study [257] provides evidence that salvaged blood in AAA surgery contains substantially higher levels of pro-inflammatory mediators than blood in total hip replacement surgery. The use of tranexamic acid in the group of patients who underwent total hip arthroplasty could have had a direct effect and attenuated inflammation due to its immunomodulatory characteristics and its direct action on white blood cells by suppressing their migration and recruitment [258,259].

The study by van der Heide H. et al. [260] investigated the pro-inflammatory dynamics of cytokines in drainage fluid in patients that underwent total knee and hip replacement surgeries. The authors detected measurable levels of all cytokines except IL-17 in the drainage fluid. Cytokine concentrations in the drainage fluid were collected at two time points: 1 h and 6 h post-procedure. IL-2, IL-4, IL-6, IL-8, IL-1β, IL-5, IL-7, IL-12, IL-13, GM-CSF, IFN-γ, TNF-α, G-CSF, and MCP-1 showed a significant increase in concentration over 6 h, with *p*-values indicating a statistically significant change (all *p* < 0.0001 except for GM-CSF, where *p* = 0.001). The levels of IL-10 showed a slight increase, but this change was not statistically significant (*p* = 0.215). Similarly, MIP-1b levels decreased slightly, but the change was not statistically significant (*p* = 0.392). IL-6 showed the most dramatic increase, increasing from 313.914 pg to 9916.640 pg. IL-4 and G-CSF levels also displayed substantial elevation, indicating a robust immune response. Other cytokines, such as IL-8 and IFN-γ, also increased markedly, reflecting typical inflammatory and immune activation following surgery [260].

In the inflammatory response observed at surgical sites, such as during total hip arthroplasty, both pro-inflammatory and anti-inflammatory cytokines are typically induced. A notable aspect of this response involves the induction of the anti-inflammatory cytokine IL1RN in HD neutrophils, which plays a role in counteracting the effects of the pro-inflammatory cytokine IL-1 by inhibiting its receptor binding [261,262]. IL-1β, known for its role in inflammation and its capacity to enhance pain sensitivity at nerve terminals, has been reported to increase post-THA [263,264]. The subsequent increase in IL1RN following THA is understood as a natural mechanism to mitigate the effects of IL-1β, potentially facilitating the involvement of neutrophils in postoperative recovery. This is supported by studies where chronic IL1RN treatment, or its recombinant analog anakinra, was shown to decrease basal nociceptive sensitivity in animal models and clinically reduce joint inflammation in rheumatoid arthritis patients [265,266,267,268,269,270,271,272]. These findings suggest that supplemental exogenous IL1RN post-THA might be beneficial in managing pain and inflammation without negatively affecting wound healing, although further research is needed to explore this possibility.

In a study [273] examining a microarray of hip-drain neutrophils, it was discovered that several cytokine genes exhibited significant changes after surgery. Specifically, interleukin-1 receptor antagonist (IL1RN), interleukin-18 receptor 1 (IL18R1), macrophage migration inhibitory factor (MIF), and macrophage inflammatory protein 3α (CCL20) were upregulated, whereas interleukin-8 receptor β (IL8RB/CXCR2) was consistently downregulated when compared to presurgery blood neutrophils. These findings were further validated using reverse transcription-polymerase chain reaction. These results suggest that there is a unique cytokine gene expression profile in neutrophils at the surgical wound site 24 h after THA surgery when compared to presurgery circulating neutrophils. By understanding these changes, we may be able to manipulate neutrophil activity to reduce postoperative pain and inflammation without compromising wound healing [273].

## 11. Materials and Methods

### 11.1. Literature Search Strategy

**Figure 1:**
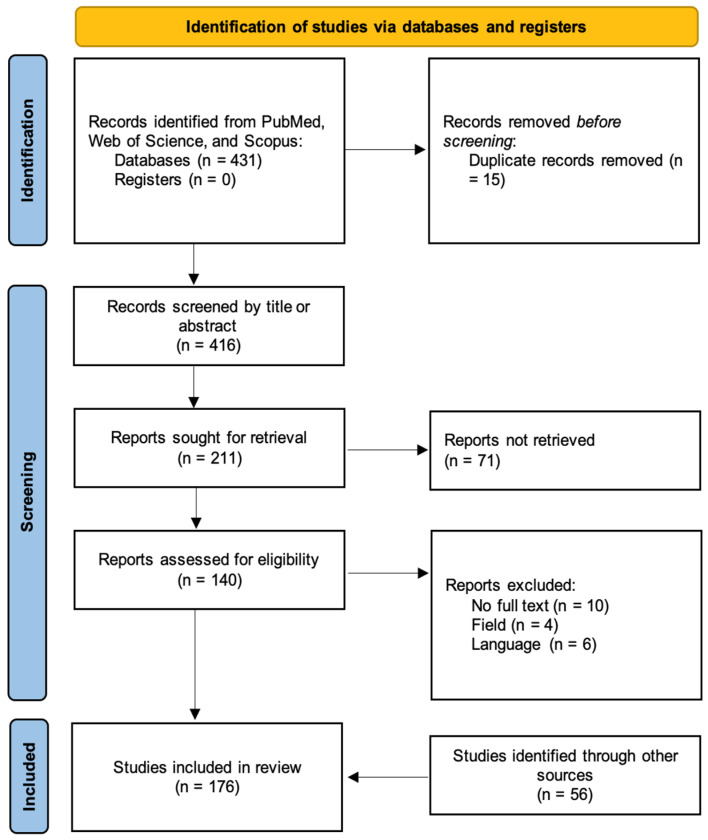
Flow diagram of included studies in the article.

To gather relevant literature for this narrative review, we conducted a comprehensive search of electronic databases including PubMed, Web of Science, and Scopus. The search terms used included “IL”, “interleukin”, “Tumor Necrosis Factor-α”, “TNF-α”, “FICB”, “Fascia iliac compartment block”, “pain”, “pain management”, “Plasminogen Activator Inhibitor-1”, “inflammatory cytokines”, “postoperative inflammation”, “surgical outcomes”, “total joint arthroplasty”, “TJA”, “THA”, “total hip arthroplasty”, “Total knee arthroplasty”, and “TKA”. We also used Boolean operators (AND, OR) to combine these terms in various ways to ensure broad and thorough retrieval of the literature. The flow diagram for the selected articles to be included in the narrative reviews is highlighted in Figure 1.

### 11.2. Inclusion and Exclusion Criteria

We included studies that specifically examined the roles and interactions of inflammatory markers in orthopedic surgical procedures and their influence on postoperative recovery, including pain management and inflammation. The review focused on both human and animal studies published in English to date (1 February 2024). Articles that did not directly assess the cytokines of interest, editorials, opinion pieces, and studies were not available in the full text were excluded.

### 11.3. Data Extraction

Both authors independently extracted the data from the selected studies. The extracted information included authors, year of publication, study design, sample size, surgical context, methods of cytokine assessment, main outcomes related to inflammation and pain, and the roles of biomarkers, as reported in the studies. Discrepancies between authors were resolved through discussion, and if necessary, an external reviewer was consulted.

### 11.4. Data Synthesis

Given the narrative nature of this review, data were descriptively synthesized. We categorized the information based on cytokine type, effects on pain and inflammation, and differences observed between clinical and experimental settings. Trends, common findings, and notable contradictions in the literature are highlighted to provide a comprehensive overview of the current understanding of these cytokines in orthopedic surgical contexts.

### 11.5. Ethical Considerations

As a narrative review, this study did not involve direct interactions with human participants or animals; thus, ethical approval and patient consent were not required.

## 12. Conclusions

Based on the extensive overview of interleukins, PAI-1, CRP, and TNF-α in inflammatory responses during the perioperative period of joint arthroplasty, several critical conclusions emerge. Pro-inflammatory cytokines like IL-1β, IL-6, and TNF-α, primarily produced by activated macrophages, play crucial roles in initiating and perpetuating inflammatory responses that exacerbate postoperative pain, directly influencing nociceptors and chronic pain development. Conversely, anti-inflammatory cytokines such as IL-10 mitigate these effects by suppressing inflammatory mediators, presenting therapeutic targets for effectively controlling pain and inflammation. The balance between these cytokines is pivotal for optimal immune response and tissue healing, impacting patient recovery and quality of life. Elevated IL-6 levels correlate with increased pain, suggesting that interventions modulating cytokine levels could enhance pain management and reduce reliance on systemic analgesics.

Despite the known roles of these cytokines, the specific contributions of PAI-1 in this context remain unclear, necessitating further research to understand its role in postoperative inflammation and pain management. Additionally, while fascia iliac compartment block (FICB) is a recognized method for managing postoperative pain, data on the concentrations of anesthetics used and their effects on interleukin trends are limited, hindering the optimization of FICB protocols. Understanding the precise impacts of anesthetic concentrations in FICB on cytokine levels is essential for refining pain management strategies.

This comprehensive understanding underscores the complexity of inflammatory processes in joint arthroplasty and highlights the potential for targeted therapeutic interventions. By exploring these aspects, future studies can optimize perioperative care, enhance surgical outcomes, and pave the way for more effective and personalized approaches to managing inflammation and pain in surgical patients. Further research into the roles and interactions of these cytokines and anesthetic techniques is crucial for advancing therapeutic strategies and improving patient outcomes in joint arthroplasty.

## Figures and Tables

**Table 1:** Effects of cytokines on pain.

Cytokines	Source	Receptors	Antinociceptive Properties	Pronociceptive Properties
Interleukins				
IL-1β	Mast cells, Schwann cells, Macrophages, Microglia, Astrocytes [213]	IL-1R1/R2/R2	Neuromodulator of LTP, acts against infections and regulates inhibitory neurotransmission [214]	Increases neuronal sensitization/mechanosensitivity of C fibers/transient receptor potential cation channel subfamily V type 1 receptors expression in dorsal root ganglia/release of proinflammatory cytokines [213,214,215]
IL-6	Macrophages and Monocytes [213]	IL-6R, sIL-6R, gp130	Regenerative processes [213]	Recruitment of mononuclear cells, T cells apoptosis inhibition, increased transient receptor potential cation channel subfamily V type 1 in dorsal root ganglia [213,216]
IL-10	Mast cells, T and B cells, Macrophages [213]	IL-10R1/R2	Immunosuppressive activity of proinflammatory cytokines release, increased release of anti-inflammatory cytokines, increases spinal microglial expression of β-endorphin [217,218]	Increases activation and proliferation of immune cells, increases INF-γ production, inhibition of the suppression of B cells [213]
Il-17	T cells, and Fibroblasts [213]	IL17RA	Anti-inflammatory effect, protection against bacterial-inflammation-induced bone loss [219]	Increases transcription of cytokines, activates nociceptors and induces hyperalgesia via neutrophil infiltrations [213]
Il-18	Macrophages, monocytes, Microglial and Astrocytes [213]	IL-18R	-	Increases hyperalgesia after intrathecal injection, induces astroglia activation and mediates microglia/astrocytes/neurons interactions [213,220]
Tumor necrosis factor				
TNF-α	Astrocytes, Microglia and Macrophages [213]	TNFR1/R2	Nerve demyelination via TNFR1 signaling	Increases neuronal sensitization and CGRP release and stimulation of oligodendrocytes regeneration [213]
Interferon				
INF-γ	CD4+ T cells, Astrocytes and Microglia [213]	INF-γR	Neuroprotective role and regulation of immunity [221,222]	Recruitment and activation of microglia, and increased excitatory synaptic transmission [213]
Chemokines				
CCL2/MCP-1	Macrophages and Monocytes [213]	CCR2	Global suppressive effects on T-cell trafficking and differentiation [218,222]	Activation of microglia, increases activity of NMDA receptors in dorsal horn neurons and of the recruitment of macrophages [223]
CXCL1/GRO-α	Macrophages and Astrocytes [224]	CXCR2	-	Involved in astroglia–neuronal interaction, central sensitization via NMDA receptors activity and attracts polymorphonuclear cells toward inflammatory sites [223]
PAI-1	Endothelium	LRP1	activates macrophages and increased pro-inflammatory cytokines [225]	
